# Sensory Ecology of Predator-Induced Phenotypic Plasticity

**DOI:** 10.3389/fnbeh.2018.00330

**Published:** 2019-01-18

**Authors:** Linda C. Weiss

**Affiliations:** Department of Animal Ecology, Evolution and Biodiversity, Ruhr University Bochum, Bochum, Germany

**Keywords:** phenotypic plasticity, daphnia, protocerebrum, deutocerebrum, chemoreceptors, neckteeth, inducible defenses

## Abstract

Ecological communities are organized in trophic levels that share manifold interactions forming complex food webs. Infochemicals can further modify these interactions, e.g., by inducing defenses in prey. The micro-crustacean *Daphnia* is able to respond to predator-specific chemical cues indicating an increased predation risk. *Daphnia* shows plastic responses by adapting its morphology, behavior, and physiology, increasing organism, and population fitness. This stabilizes community structures. This review will describe the progress that has been made in understanding the high degree of plasticity observed in the model crustacean *Daphnia*. I summarize current knowledge on the processes of predator detection, ranging from the nature of biologically active chemical cues to the underlying neurophysiological mechanisms. With this, I aim to provide a comprehensive overview on the molecular mechanisms of *ad hoc* environmental phenotypic adaptation. In times of climate change and pollution understanding information transfer in aquatic systems is valuable as it will allow us to predict whether and how community structures are being affected.

## Introduction–Sensory Systems

Organisms have evolved the capacity to respond to changes in the environment by phenotypic adaptation. Fluctuations in the biotic environment are dominated by the ever-changing presence and absence of con- and heterospecific organisms, prey, and predators. Therefore, detecting mates, prey or predators, and responding appropriately is critical for organisms' survival. Species have evolved dedicated sensory systems that are optimized to their particular ecology. Chemical perception is of central importance, alongside vision, mechanoreception, and electrical senses (Wisenden, 2000; Ferrari et al., 2010). Chemical cues are of particular relevance in aquatic environments as they can be identified regardless of turbidity and are reliable in space and time. Many prey organisms are able to detect the presence of predators even against a background of high chemical diversity. This allows the prey to respond to predator presence with plasticity of their phenotype and increase survival chances. Yet, for any kind of plasticity to be adaptive in the context of an increased risk of predation, the correct interpretation of the environmental challenge is pivotal, as a mal-adapted phenotype could in fact increase its predation risk. Understanding the sensory mechanisms and neuronal pathways underlying predator perception is also critical in the light of anthropogenic disturbances, which often interfere with neuronal signaling cascades.

This review focuses on the neuronal mechanisms underlying predator-induced morphological plasticity. Other defensive strategies like behavioral and life history adaptations, have so far received less attention. I will introduce the concepts of phenotypic plasticity and inducible defenses and explain their ecological relevance. I summarize the general insights that underlie chemical predator perception, neuronal wiring, neurophysiology, and neuronal plasticity.

This review centralizes on the perception and neuronal processing of kairomones. In the classical and broad sense kairomones are defined as interspecific chemical cues perceived by a benefiting prey to reduce the negative impact of a natural enemy (Ruther et al., 2002). As there are already numerous, valuable, and detailed reviews on the perception of chemical alarm pheromones (Sorensen and Stacey, 2004; Døving and Lastein, 2009; Dew et al., 2014; Ahuja et al., 2015; Lastein et al., 2015; Wisenden, 2015), these and the perceptive mechanisms will not be reviewed here.

I detail current knowledge of predator detection and neuronal signaling of predator-induced morphological plasticity in the freshwater crustacean *Daphnia*. This zooplankter is an important component of freshwater food webs, showing a strong plasticity against a range of predators. Several *Daphnia* genomes have now been sequenced (Colbourne et al., 2011; Ye et al., 2017, www.fleabase.org) and accordingly molecular biology applications (e.g., *in situ* hybdridization, RNAi, etc.) are becoming available (Kato et al., 2012; Nakanishi et al., 2014; Naitou et al., 2015; Nong et al., 2017). Many studies have investigated the changes in gene expression levels that affect morphological defense expression in *Daphnia pulex* (Schwarzenberger et al., 2009; Spanier et al., 2010; Miyakawa et al., 2015; Rozenberg et al., 2015, 2016; Hales et al., 2017), while only a few have looked at the (neuro-) physiological changes (Hanazato, 1991; Barry, 1999, 2002; Weiss et al., 2012a; Miyakawa et al., 2015).

## Phenotypic Plasticity

Genotypes equipped with adaptive strategies to increase individuals' fitness can help organisms to conquer environments with fluctuating conditions. From an ecological and evolutionary perspective, phenotypic plasticity is a powerful, and widespread means of organismal adaptation. Phenotypically plastic organisms may respond to environmental extremes, and Bradshaw was one of the first to recognize the importance of genetic variation of plasticity in an evolutionary context (Bradshaw, 1965). She postulated that plasticity should be understood as a trait that underlies evolutionary trajectories, such as selection. Moreover, phenotypic plasticity can be discussed as a means of evolution affecting biodiversity by enabling better niche use or the exploitation of several niches. Evolution of adaptive phenotypic plasticity has led to the success of organisms in novel habitats, and potentially contributes to genetic differentiation and speciation (Miner et al., 2005; West-Eberhard, 2005; Theißen and Melzer, 2016). An expanding body of work examines how plasticity can affect all levels of ecological organization through effects on demographic parameters, but also through direct and indirect species interactions, such as competition, predation, and coexistence (reviewed in Miner et al., 2005). For example, there are recent arguments that predator-induced plasticity makes evolutionary change possible and that evolution may be preceded by genetic assimilation (Reger et al., 2018).

Despite the growing body of work, the question of how plasticity is realized remains and the underlying molecular mechanisms are often unexplored. To elucidate these pathways, it is important to first distinguish whether the phenotypic adaptations are induced by biotic or abiotic environmental cues. The abiotic environment can affect the phenotype by physical laws (Kelly et al., 2012). So, for example, temperature can cause phenotypic changes through enzyme kinetics and diffusion rates (Kelly et al., 2012). Similarly, low nutrient availability can impact growth and morphology. Cold acclimation is known to result in metabolic responses involving increases in mitochondrial amount and capacity (Healy et al., 2017). Other examples of abiotic induction of plasticity are photoperiod-induced life history shifts in aphids (Simon et al., 2011) and timing of metamorphosis in amphibians (Wright et al., 1988).

Biotic environmental challenges originate from con- and heterospecifics and are in general signaled through chemical cues: predators release semiochemicals (kairomones), which indicate predation risk and conspecifics may release density cues, which modify and fine tune the response (Tollrian et al., 2015). The phenotypic change is realized through a complex interaction between these environmental chemical cues and organismal sensors. For example, prey species sense the chemical cues released by their predators. The active sequence of events from cue release → cue perception → signal transmission → endocrine signaling → phenotype adaptation is mostly elusive (Beldade et al., 2011; Morris and Rogers, 2014). One particular form of phenotypic plasticity are inducible defenses, where the phenotype is adapted to an increased predation risk.

## Inducible Defenses

Predation is a major factor driving adaptation and predator-induced defenses are an intriguing form of phenotypic plasticity that can decrease the likelihood of predator encounter or detection, and reduce the effectiveness of predator attacks. Ecologically, inducible defenses have been discussed to be of high importance as they dampen predator–prey oscillations, thereby stabilizing population dynamics (Verschoor et al., 2004). Many different defensive strategies expressed against an increased predation risk have been described (reviewed in Tollrian and Harvell, 1999). Defenses may occur in the form of behavioral or morphological adaptations or shifts of life history parameters, and many taxa show a variety of different anti-predatory adaptations (reviewed in Weiss et al., 2018).

For precise development of the appropriate defense, prey organisms must be able to accurately distinguish between predators; the expression of a defense that is not effective against the predator may pose a disadvantage and thereby reduce organism fitness (reviewed in Weiss and Tollrian, 2018). In addition, prey organisms must also be able to respond to multiple predators simultaneously as in most ecosystems predation is not limited to a single predator. All this is further complicated, as the expression of inducible defenses is not simply adapted to the predator but also to the predation risk (Tollrian et al., 2015; Crane and Ferrari, 2017). It was shown that, with growing conspecific numbers conspecific chemical cues indicate that the predation risk decreases, upon which defense expression is reduced (Peacor, 2003; Tollrian et al., 2015).

The fact that organisms are able to distinguish between predators, con- and heterospecifics and perform predation risk assessment in combination with a fine-tuned defense expression, shows that dedicated sensory systems must have evolved, enabling correct interpretation of the environment. The freshwater crustacean *Daphnia* has been especially well-studied for its capacity to express a diversity of inducible defenses.

## Inducible Defenses in *Daphnia*

**Figure 1:**
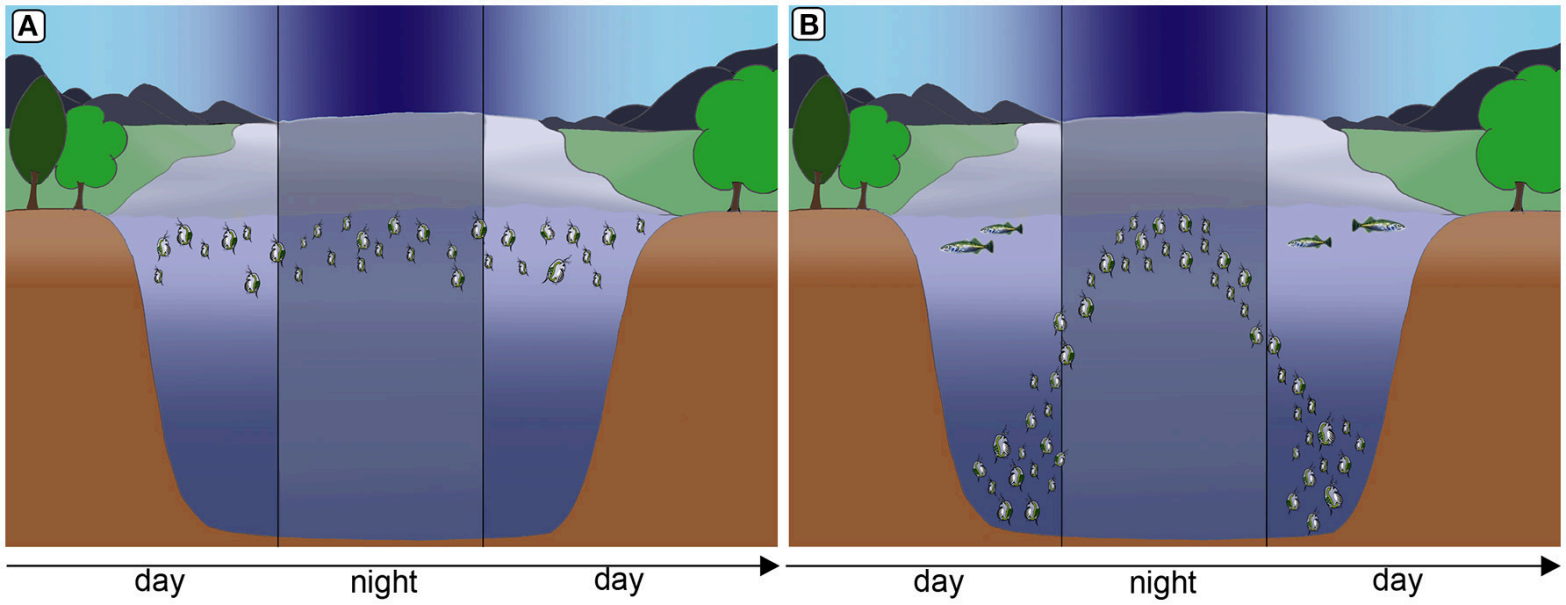
Diel vertical migration as a form of behavioral defense in *Daphnia* to counter fish predation. In the absence of fish predation *Daphnia* remain in warm, nutritious water strata **(A)**. Under predation, *Daphnia* migrate to deeper water strata, seeking refuge from visual predators. However, during the night they migrate to nutrient rich and warmer strata for feeding **(B)**. Images by Weiss.

Inducible defenses in *Daphnia* are manifold. Some *Daphnia* species show behavioral adaptions to fish predation (Figure 1). They perform fish-induced diel vertical migration, seeking refuge in the deeper water strata during the day, in order to escape visual predators (Ringelberg, 2010). Intriguingly, this defensive strategy is induced by two co-occurring cues: the predator and light conditions. Also, reduced swimming speeds have been reported to occur countering predation by the tactile predator the phantom midge larvae *Chaoborus* (Dodson et al., 1997). Shifts of life history parameters, are similarly expressed against fish predation (Brett, 1992; Hanazato et al., 2001, Figure 2; Boersma et al., 2009). Here somatic growth is traded with reproduction and the presence of fish induces early maturation at a smaller size and the production of more and smaller offspring in subsequent generations (Brett, 1992; Tollrian, 1995; Carvajal-Salamanca et al., 2008; Boersma et al., 2009).

**Figure 2:**
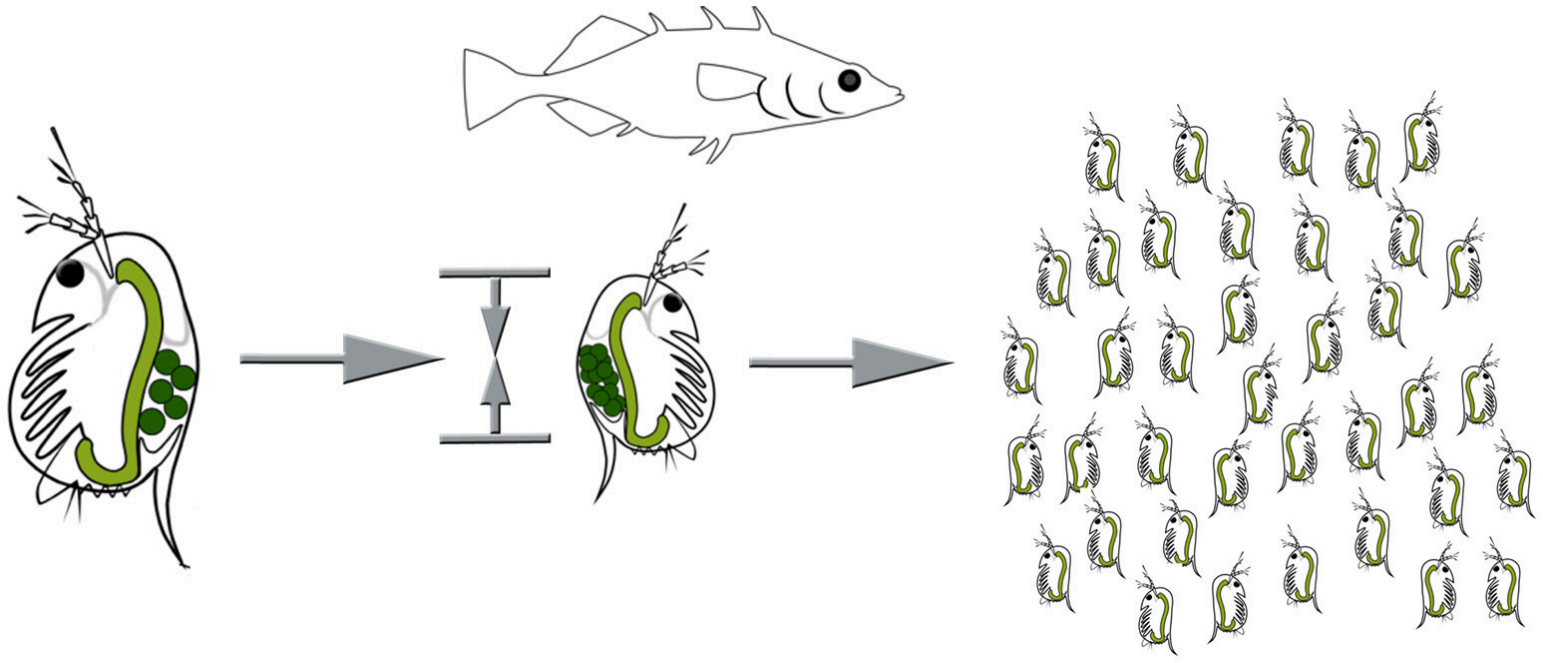
Life history adaptations under fish predation. Resources are shifted from somatic growth to reproduction, resulting in earlier sexual maturity at a smaller size, and an increased number of smaller offspring. Images by Weiss.

Morphological defenses in *Daphnia* have been in the focus of ecological research for decades (Krueger and Dodson, 1981; Tollrian, 1993; Stollewerk, 2010; Tollrian and Leese, 2010; Weiss et al., 2012b); including helmet development in *D. cucullata* (Tollrian, 1990, Figure 3A) and neckteeth expression in *D. pulex* against *Chaoborus* spec. predation (Krueger and Dodson, 1981; Tollrian, 1993; Weiss et al., 2016, Figure 3B), crest development in *D. longicephala* against the heteropteran backswimmer *Notonecta* spec. (Grant and Bayly, 1981; Weiss et al., 2015, Figure 3C), head- and tail-spine development in *D. lumholtzi* against fish predation (Tollrian, 1994, Figure 3D) and crowns of thorns in *D. atkinsoni* against the tadpole shrimp *Triops* spec. (Petrusek et al., 2009).

**Figure 3:**
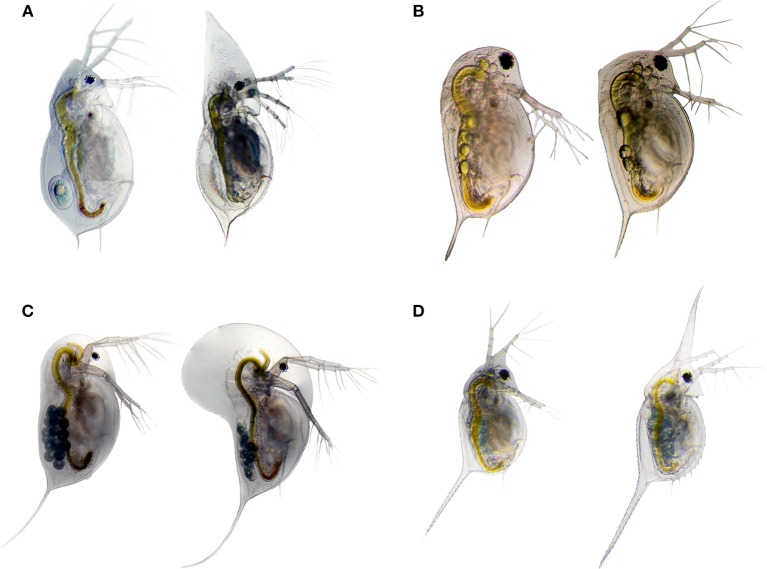
Inducible morphological defenses are manifold in the genus *Daphnia*. The listed examples show helmet expression in *D. cucullata* **(A)**; crest expression in *D. longicephala* **(C)**; head- and tail-spine formation *D. lumholtzi* **(D)**, and neckteeth expression in *D. pulex* **(B)**. Undefended morphotypes are displayed on the left side, and the defended morphotype on the right side. Images by Becker & Weiss.

## Predator-Specific Chemical Cues–Identification of Friends and Foes

Sensory information often plays a pivotal role in shaping species interactions (Hay, 2009). Species acquire information about their biotic and abiotic environment by detecting specific chemical cues (Atema et al., 1988). Organisms with poor vision and those in turbid environments are particular beneficiaries of chemical cues. Furthermore, chemical cues can be transmitted over long temporal, and spatial scales (Wisenden, 2000). A network of chemical cues is speculated to significantly complicate our current knowledge of trophic interactions (Burks and Lodge, 2002; Pohnert et al., 2007). It is therefore obligatory to not only describe trophic interactions *per se* but also identify the modifying agents. The different type of cues are distinguished based on their origin; so that the sources need to be determined prior to correct categorization into either alarm cues or predator specific cues (Hazlett, 2011; Wisenden, 2015). Sometimes, this even requires the chemical identification of the compound(s). While alarm cues are chemicals of conspecific prey, and released upon mechanical damage of the same, predator specific cues are generally unintentionally released by the predator. Predator cues can also be but do not have to be associated to foraging activities (Mitchell et al., 2017). While there are numerous reviews on alarm cues (Hazlett, 2011) some with the special focus on fish (Døving and Lastein, 2009; Lastein et al., 2015; Wisenden, 2015), predator specific cues are less well-reviewed and will therefore be the focus here.

Interspecific chemical cues released by predators induce the development of defensive features in prey organisms (Tollrian and Harvell, 1999; Weiss et al., 2012b). These, so-called, kairomones are released by a sender serve as an advantage for a receiver, as in this context they indicate an increased predation risk. Kairomones decrease the efficiency of the predator (Jeschke et al., 2002) thereby affecting the dynamics of entire food webs (Kats and Dill, 1998; Dicke and Grostal, 2001; Verschoor et al., 2004; Vos et al., 2006).

When these substances are associated with foraging activities, the value of this cue as a signaling agent increases significantly for the prey organisms and evolution of sensitivity to such cues should be favored. Unfortunately, many of the infochemicals's structure and/or composition remain elusive. Kairomones have disparate chemistry, for example they can be proteins that signal the development of inducible defenses in ciliates (Kusch and Heckmann, 1992), which develop lateral wings in the presence of the predatory ciliate *Lembadion*. Alternatively, aliphatic sulfates released by *Daphnia* inducing defensive coenobia in algae (Yasumoto et al., 2005), and composites like the recently described copepodamides induce the production of the paralytic shell fish toxin in the dinoflagellate *Alexandrinum minutum* (Selander et al., 2015). Trigonelline and homarine were shown to induce fear in mud crabs (Poulin et al., 2018). In rodents, fear responses are induced by volatile molecules such as TMT, 2-PEA, and 2-PT, which result from meat digestion in the predator (Pereira and Moita, 2016).

These signals appear to be enormously diverse in chemical and temporal structure, and the ability to identify and quantitate them, especially from the aquatic environment, has been a major challenge in chemical ecology. The identification of such signaling agents is however a pivotal component in our understanding of predator perception. Of course, chemoreceptors play a critical role, where the identification and deorphanization of an explicit chemoreceptor requires knowledge of the ligand.

## The *Chaoborus* Kairomone

Fourth instar phantom midge larvae of the genus *Chaoborus* (Diptera) prey upon juvenile *D. Pulex*. The larvae grasp the daphniid prey with their feeding basket composed of head appendages. The prey is captured and consumed by alternating movements of the mandibles. All digestible parts are consumed, and the indigestible components are egested. The kairomone is released during the egestion process. It comprises a family of compounds consisting of long-chained (≥C14) fatty acids coupled to the N-terminus of an L-glutamine residue. Lipidated -L-glutamine conjugates released by feeding *Chaoborus* larvae trigger defensive neckteeth formation in juvenile *Daphnia* (Weiss et al., 2018).

These molecules carry characteristics of suitable aquatic infochemicals: they are water-soluble and thus pass from the emitting organism (i.e., predator) to the receiving organism (e.g., prey). Additionally, because of their origin from active digestion they are good indicators of predation risk.

Regarding the origin of the kairomone, it is anticipated that the larvae take up fatty acids from their diet (in this case the *Daphnia*, yet *Chaoborus* also consumes e.g., ciliates and this rearing medium also induces defensive neckteeth, Tollrian personal communication) and use them for glutamine assimilation in the mid-gut as seen in many caterpillars or other dipteran species like *Drosophila* (Mori and Yoshinaga, 2011; Yoshinaga, 2016). Glutamine seems to function as a nitrogen storage as it is mandatory for many biosynthetic pathways. This also explains why co-evolution leading to suppression of chemical release is hampered. Unsurprisingly, substances whose production cannot be avoided are exploited as reliable interspecific information cues by the different prey species. The active components of the *Chaoborus* kairomone are only produced during active feeding, however the type of diet is irrelevant (Tollrian, personal communication). For categorization purposes, this means that the *Chaoborus* kairomone is not a dietary alarm cue, but an activity dependent compound produced by the predator during digestion. It is beneficial for the predator only in the context of metabolism. A benefit in the context of information transfer is only advantageous for the receiver, i.e., the prey.

Having the information of the inducing agent is critical for the identification of respective chemoreceptors and the signaling pathways used to detect such cues within environmental noise.

## Chemoreceptors

By 1991 Buck and Axel had identified a set of ca. 1,000 olfactory receptors (ORs) in rats (Buck, 1996). These belong to a class of rhodopsin-like G-protein coupled receptors (GPCRs) and have been identified in all vertebrates. No homologs of these receptors have been detected in any protostome. In insects, a distinct group of ORs and gustatory receptors (GRs) have been described that are not homologs to the vertebrate ORs (Vosshall et al., 1999). They represent an individual gene family, with the GRs being the ancestral type. These are ionotropic receptors (ligand-gated ion channels), similar to traditional ionotropic glutamate receptors such as kainite, N-methyl-D-aspartate (NMDA), and AMPA receptors. Compared with vertebrate ORs, insect ORs show an inverted membrane topology with extracellular carboxyl and intracellular amino terminals (Galizia and Sachse, 2010). Insect ORs function as heteromultimers composed of at least one ligand-specific OR and the co-receptor Orco (Vosshall et al., 1999; Galizia and Sachse, 2010; Wicher, 2018). Neither Orco nor ORs are present in the genome of the crustacean *D. pulex*, indicating that ORs are insect specific. However, GRs were found in *Crustacea*, just as in insects (Peñalva-Arana et al., 2009).

Another example of chemoreceptor proteins is a subset of ionotropic receptors (IRs) found in all protostome clades, known as the IRs. These probably evolved from the non-NMDA ionotropic glutamate receptors in ancient protostomes (Croset et al., 2010).

For a comprehensive review of the different chemoreceptor proteins and their evolution please refer to (Vosshall et al., 1999; Derby et al., 2016; Brand et al., 2018).

Our understanding of how predator kairomones are perceived and processed is very limited and dedicated chemoreceptors have predominantly been identified in mice (Isogai et al., 2011). In this study, con- and heterospecific chemical cues stimulated neurons in the vomeronasal organ (VNO) were screened for chemoreceptor expression (Isogai et al., 2011). It was thus possible to detect the activation of neurons with specific receptors during chemical cue exposure. The authors found that within the 250 receptors that are expressed in the VNO of mice, 71 receptors in total exclusively respond to heterospecific cues, only 11 of these receptors also responded to conspecific cues (Isogai et al., 2011). Intriguingly, some receptors were exclusively responsive to different types of predators so that two receptors responded to snake cues, while others responded to owl cues. Each predator species tested activated a distinct subset of receptors, pointing to the capacity of mice to be able to distinguish between predators (Isogai et al., 2011).

In general, chemical cues bind to chemoreceptors that are located on some kind of chemoreceptive organ innervated by olfactory receptor neurons (ORNs).

Olfactory wiring is achieved as one ORN expresses one functional receptor type, and all ORNs expressing the same receptor type coalesce in one glomerulus of the odor information processing region e.g., the olfactory lobes in arthropods (Strausfeld and Reisenman, 2009), or the olfactory bulb in mammals (Nagayama et al., 2014).

## Chemoreceptors in *Daphnia*

Compared with other taxa (like insects and mammals) not much is known about chemoreceptors in *Daphnia*. There is a known repertoire of 58 chemoreceptors that cluster in 3 distinctive superfamilies and share sequence homology with insect gustatory receptors (Grs). No genes encoding proteins similar to the insect odorant receptors (Ors) were found, which might indicate a quite recent expansion of this gene lineage concomitant with the evolution of hexapods (Peñalva-Arana et al., 2009). This is further supported by the observation that, as yet, Ors are also absent in other crustacean genomes. Yet, it is highly questionable that *Daphnia* relies on these 58 Grs only. Recently, it was reported that crustacean olfactory receptors are orthologs of insect olfactory IRs and the *Daphnia* genome holds an abundant number of IRs (Croset et al., 2010). In fact, it was shown later in lobsters that two IR subunit genes *PargIR25a* and *PargIR93a* are expressed in most or all spiny lobster ORNs, as confirmed by *in situ* hybridization (Corey et al., 2013). Other encoded IR subunits are expressed only sparsely, suggesting an ORN-specific expression pattern. This suggests that, as in insects, the odorant specificity of individual lobster ORNs is determined by a specific set of expressed subunits and that these subunits are composed of *IR25a* and/or *IR93a* co-receptors (Croset et al., 2010; Corey et al., 2013).

Thus, it is likely that IRs play a general role in initiating chemosensory signaling in crustaceans and also in *Daphnia*. Yet, limited attempts have been undertaken for heterologous expression and deorphanization of the chemoreceptors that have been identified by sequence homology with other arthropods. It is also likely that there are additional classes of chemoreceptors yet to be discovered (Derby et al., 2016; Harzsch and Krieger, 2018).

For a precise description of how environmental chemical cues are decrypted, it is pivotal to deorphanize receptors specific for kairomones but also disentangle the underlying neuronal structures involved in kairomone perception such as nervous fibers and the computational steps in higher brain areas.

## Neuronal Wiring of Olfactory Receptor Neurons

Neural circuits are both anatomical and functional entities and the involved neurons never function in isolation. Such neuronal circuits process specific kinds of information and their identification is crucial for the understanding of how sensory information is encoded. There is a general concept of odorant coding found in vertebrates and invertebrates. Odorants are first detected by chemoreceptors located on ORNs. The axons of the neurons then organize centrally into glomeruli organized by olfactory receptor type (Vosshall et al., 1999; Derby et al., 2016; Harzsch and Krieger, 2018). A glomerulus is a roundish substructure that contains most synapses within the antennal lobe or olfactory bulb (depending on taxon). In vertebrates and invertebrates alike, projection neurons (PNs) relay olfactory inputs to higher-order brain areas like the mushroom bodies and the lateral horns (in insects). These brain areas permit associative learning or mediate innate behaviors (reviewed in Galizia and Rössler, 2010).

While the projection neurons are uni- and multi-glomerular and suspected to be of cholinergic pharmacology (Galizia and Rössler, 2010), there are also local neurons (LNs) that interconnect the glomeruli and are of the amacrine type (Homberg et al., 1989). They are diverse in morphology and controlled by inhibitory neurotransmitters like GABA or excitatory neurotransmitters like acetylcholine. For further detail please see Galizia and Rössler (2010); Galizia and Sachse (2010); Derby et al. (2016); Harzsch and Krieger (2018).

## Neuronal and Cellular Wiring in *Daphnia*

**Figure 4:**
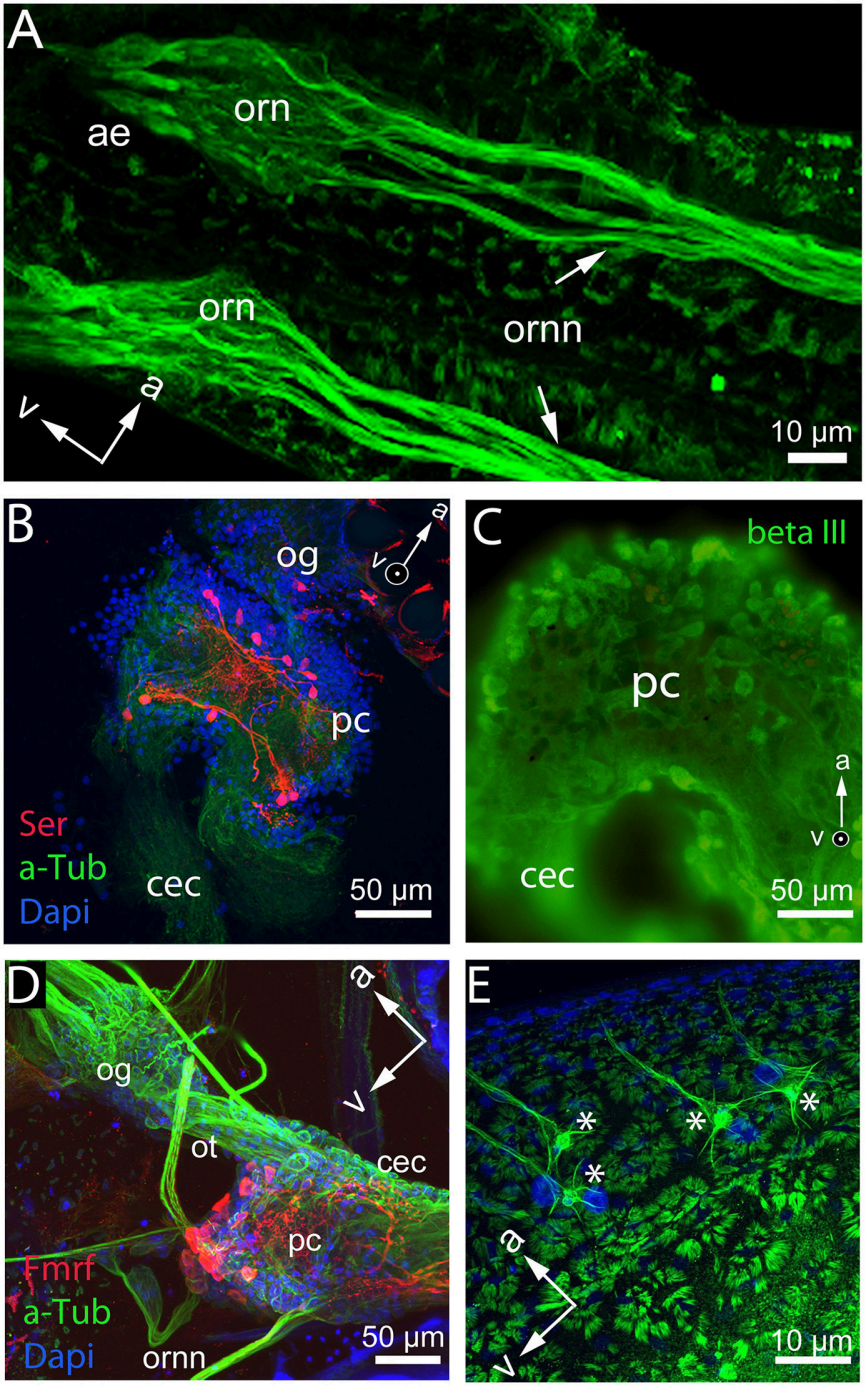
Whole mount and brain mount preparation of the *Daphnia* nervous system displaying differential functional qualities of cells. **(A)** Base of the antennules and cuticular insertion of the aesthetascs (ae) with olfactory receptor neurons (orn) and olfactory receptor neuron neurites (ornn) extending from the first antennule to the deutocerebrum of the brain in *D. longicephala*. **(B)** Protocerebrum (pc) and optic ganglia (og) of *D. magna* show neurons stained with anti-serotonin antibody (red). Cytoskeleton is stained with anti-alpha tubulin (green) and nucleoli with dapi (blue). **(C)** Protocerebrum overview of the *D. longicephala* brain stained with an antibody detecting neuron-specific beta III tubulin. **(D)** Whole mount preparation of a *D. longicephala* head displaying the optic ganglia (og) connected via the optic tract (ot) with the protocerebrum (pc) extending into the circumesophageal connective that connects to the tritocerebrum (not shown). The olfactory receptor neurons connect to the central brain via the deutocerebrum. Nucleoli are stained in dapi displayed in blue, the cytoskeleton is stained with anti-alpha tubulin in green, and red displays cells with anti-Fmrfamide reactivity. **(E)** Polyploid cells (asterisks) lining the region of crest expression in *D. longicephala* stained with anti-alpha tubulin. Cells are characterized by a large nucleus stained with dapi (blue), lateral extensions, and one dominant extension innervating the epidermal cells of the crest. Orientation of the preparations is marked by arrows in the anterior (a) and ventral (v) directions. Images by Ioannidou & Weiss.

In order to understand the cellular mechanisms of plasticity, an overview of the overall nervous system and the functioning of the individual components is necessary. Even if the neuroanatomy of the *Daphnia* nervous system appears comparatively simple, it is known to be able to discriminate a vast array of intra- and interspecific signals. The *Daphnia* brain is of classical arthropod organization and consists of three regions, the protocerebrum, the deutocerebrum, and the tritocerebrum (syncerebrum) as described in other branchiopod crustacean species (Harzsch and Glötzner, 2002; Kirsch and Richter, 2007; Fritsch and Richter, 2010; Kress et al., 2016). In the protocerebrum, the optical neuropils are connected via the optical tracts with its remaining scaffold (Weiss et al., 2012c). The deutocerebrum receives nerve fibers from the antennule (Weiss et al., 2012c, Figure 4A). Nerves originating from the tritocerebral ganglia enter the antenna, the labrum, and the alimentary tract (Weiss et al., 2012c). The tritocerebrum is thus also involved in functions of the stomatogastric nervous system (Heribert, 1915; Bullock, 1965; Weiss et al., 2012c). The protocerebrum is the anterior-most neuropil and comprises the largest portion of the brain (Figures 4B–D). The deutocerebrum is proximal to the protocerebral neuropil. The deutocerebral neuropil is less distinctive than the protocerebrum and consists of a pair of undifferentiated neuropils (Hallberg et al., 1992; Harzsch, 2006). The question now is, how are such adaptive processes encoded on the neuronal level. The receptors for the detection of predator cues were shown to be located on the first antennae (Weiss et al., 2015, Figure 4A). From here neurites extend to the deutocerebrum of the brain (Weiss et al., 2012c, Figures 4A,D). Yet, olfactory glomeruli in the deutocerebrum have not been detected Hallberg et al., 1992; Weiss et al., 2012c and the precise wiring underlying predator detection in *Daphnia* is unknown.

Likewise, the chemical and functional description of individual brain cells and specialized brain centers is yet understudied. For example, there is a group of serotonergic cells located in the protocerebrum that probably control phototactic behaviors (Rivetti et al., 2018, Figure 4B) and may therefore also be involved in predator-induced diel vertical migration patterns, which are triggered by changes in light intensity (Ringelberg, 2010).

On the cellular level outside the nervous system, a group of large polyploid cells located in close association with the morphological defense structure in various daphniid subgenera was suggested to be involved in the development of defensive traits (Figure 4E). These cells were speculated to serve as central control stations secreting proliferation agents (Beaton and Hebert, 1997) like dopamine inducing the mitotic activity in the vicinity of these cells (Weiss et al., 2015). Nonetheless, it remains only speculative how these cells are controlled and how they determine the development of phenotypically plastic morphological defenses.

## Neurophysiology of Predator-Induced Defenses in *Daphnia*

Neurophysiological stimulation studies have shown that the cascade underlying predator perception and defense expression, comprises multiple signaling components, including the involvement of cholinergic, glutaminergic and GABAergic signaling (Weiss et al., 2012a; Miyakawa et al., 2015). In general, acetylcholine in the brain alters neuronal excitability, influences synaptic transmission, induces synaptic plasticity, and coordinates firing of groups of neurons (Picciotto et al., 2012). As a result, it changes the state of neuronal networks throughout the brain and modifies their response to internal and external inputs, which is the classical role of a neuromodulator (Picciotto et al., 2012). Glutamate is a dominant neurotransmitter in nervous systems and activates neurons (Meldrum, 2000). Neuronal receptors for glutamate are divided into two groups: the metabotropic glutamate receptors, which are members of the G-protein coupled receptor family, and ionotropic glutamate receptors, which are members of the ligand-gated ion channel family. Ionotropic glutamate receptors are further divided into three groups whose names are derived from specific agonists: NMDA-type, (±)-α-amino-3-hydroxy-5-methyl-4-isoxazole-propionic acid (AMPA)-type and kainate-type (Meldrum, 2000). These subtypes are expressed mainly in central nervous systems and are involved in various biological processes, including memory and learning, in many animal species (Malenka and Nicoll, 1999). Genes coding for these receptors were identified using microarrays and their involvement in defense expression was further validated with a functional analysis using the receptors' antagonists (Miyakawa et al., 2015).

GABA is a major inhibitory neurotransmitter, reducing a nerve cell membrane potential and thereby decreasing its excitability (Wu and Sun, 2015). GABA is always functional in the nervous system, fine-tuning neuronal responses and controlling neuronal firing rates (Wu and Sun, 2015). In predator-exposed *D. pulex*, genes for G-protein coupled GABA receptors were differentially expressed (Miyakawa et al., 2015). Only the GABA_B_ receptor type is metabotropic and therefore G-protein coupled. While Barry (2002) antagonized the actions of GABA using picrotoxin, a non-competitive antagonist of the ionotropic GABA_A_ receptor (Barry, 2002), neurophysiological stimulation with GABA did not validate direct involvement of GABergic signaling (Weiss et al., 2012a). Indirect GABAergic signaling is reasonable, but not observable with simple neurophysiological stimulation in bioassays. Rather this requires e.g., patch-clamp measurements of ion currents in culture cells, or optogenetic strategies applied *in vivo* (Spoida et al., 2014).

## Neuronal Plasticity

Ever since the pioneering work of Drs. Hubel and Wiesel more than 40 years ago, neurobiologists have appreciated that the environment plays an essential role in shaping neural connectivity. These observations are framed by the term neuronal plasticity, which is known as the ability of the brain to change throughout an individual's life. This includes changes in gray matter, constant removal and creation of synapses depending on the activity level, or dendritic outgrowth adjustment all according to neuronal activity levels. This kind of activity-dependent plasticity is a form of functional and structural neuroplasticity arising from cognition and experience. It is thus the basis for learning and the formation of memories. Also, neuronal plasticity is a result of changes in gene expression patterns triggered by dedicated signaling cascades activated by signaling molecules such as calcium, dopamine, and glutamate.

Within an ecological perspective it has been seen that both relative brain size and structure are statistically correlated with environmental parameters. These include spatial complexity of the natural and social environment, water depth, light environment, and predation (Samuk et al., 2018). For example, an analysis of 623 pairs of predator and prey species of fish found that on average, prey species tend to have larger brains than the species that prey upon them, perhaps suggesting a “cognitive arms race” (Samuk et al., 2018).

Other studies described how predator exposure can change brain morphology e.g., predator exposed nine-spined sticklebacks grow larger *bulbi olfactorius* (Gonda et al., 2011), but the opposite effect has also been observed where predator-exposed brains become shorter and narrower. The underlying cause of this change in morphology, however, was not investigated. It remains elusive, from where the structural change originates, so that an increase in brain size could indicate an increased number of nerve cells or an increased number of cellular connections.

## Neuronal Plasticity in *Daphnia*

If and how the neuroanatomy of the brain changes during predator exposure requires investigation. So far, only the involvement of NMDA receptors, which only respond on over-activation due to the magnesium block and are known to be essentially involved in long-term potentiation in associative learning, points to a degree of synaptic neuronal plasticity.

While the perception of predators is performed by the above described actions of the nervous system, also epigenetic changes are anticipated to contribute to the modification of an organism's phenotype. Such changes can also be inherited to subsequent generations rendering these better adapted to environmental conditions.

## Epigenetics of Predator Induced Defenses

Epigenetics study the emergence of different phenotypes that result from a single genotype (Bonasio, 2015). Up to date, only little attention has been paid to epigenetic modifications and how these may affect ecological interactions. Well-known changes are described by non-coding RNAs, histone modifications and cytosine methylation (Harris et al., 2012). Ultimately, all these mechanisms lead to changes in gene expression patterns, but do not change the DNA sequence itself. Epigenetic changes are of particular interest not only because they are affected by environmental conditions but also because of their heritability. This can either take place during meiosis or mitosis. During mitosis epigenetic changes are responsible for the maintenance of discrete transcriptional states and e.g., control cell identity over multiple rounds of cell division (Duncan et al., 2014; Bonasio, 2015). During meiosis epigenetic changes can be transferred to subsequent generations. So environmental changes may be epigenetically imprinted and passed on to offspring even after the initial stress has disappeared (Harris et al., 2012). The role of epigenetics in the context of defense systems and adaptive morphotypes is not yet fully exploited. Parthenogenetic organisms like *Daphnia* are discussed as valuable models for such endeavors (Harris et al., 2012; Robichaud et al., 2012), as they have the epigenetic repertoires (e.g., differential methylation Asselman et al., 2015 and histone modification Robichaud et al., 2012) as well as the explicit ability to express context dependent phenotypes within (Weiss and Tollrian, 2018) and across generations (Agrawal et al., 1999).

## Outlook

The ability of many organisms to adjust to the predation risk and to be able to distinguish between predators shows a distinct capacity to sense and interpret the environment. This is pivotal for an individual's fitness and ultimately for ecosystem stability.

In recent years, it has become increasingly clear, that many anthropogenic agents released into the environment affect the sensory systems of marine and freshwater species.

For example, many studies have demonstrated that elevated pCO_2_ levels in the oceans and in freshwater ecosystems affect organismal neurobiology (Nilsson et al., 2012; Hamilton et al., 2014; Weiss et al., 2018). In many cases this prevents the correct interpretation of the environment and can lead to inappropriate responses (Chivers et al., 2014; Weiss et al., 2018). This may render prey species more susceptible to predators, which can have cascading effects on the ecosystem level. Likewise, a number of laboratory studies suggest that anthropogenic pollutants can disrupt chemoreception, even at low, non-toxic concentrations, but there are few tests of whether real-world variation in water quality affects chemoreception (Troyer and Turner, 2015).

These observations demonstrate the necessity to further analyze chemical signaling cues together with the sensory mechanisms that mediate environmental adaptations. With next-generation sequencing strategies, genome mining for e.g., chemoreceptors is possible and also pivotal for any molecular investigations. The availability of novel genome-editing strategies (Crispr/Cas9, TALEN, RNAi) (Kato et al., 2012; Nakanishi et al., 2014; Naitou et al., 2015) in combination with optogenetic applications (Herlitze and Landmesser, 2007; Deisseroth, 2011) and electroantennograms (Simbeya et al., 2012) will allow us to further decipher the molecular mechanisms underlying predator-induced phenotypic plasticity.

## Author Contributions

The author confirms being the sole contributor of this work and has approved it for publication.

### Conflict of Interest Statement

The author declares that the research was conducted in the absence of any commercial or financial relationships that could be construed as a potential conflict of interest.
